# Supplementary material for: Increasing evidence that bats actively forage at wind turbines
Source: PeerJ. 2017 Nov 3;5:e3985. doi: 10.7717/peerj.3985 (PMC5672837; doi:10.7717/peerj.3985)
Supplement: Figure S1 — Biweekly averages and 95% CI of the proportions of each order collected during July–August malaise trapping in 2012, 2013, and 2015 at the Wolf Ridge wind farm. The “other” category includes Homoptera, Mantodea, Trichoptera, and spiders. [file peerj-05-3985-s001.docx]

**Figure S1.** **Biweekly proportions of insect orders surveyed at turbine towers.**

Biweekly averages and 95% CI of the proportions of each order collected during July-August malaise trapping in 2012, 2013, and 2015 at the Wolf Ridge wind farm. The “other” category includes Homoptera, Mantodea, Trichoptera, and spiders.
