# Supplementary material for: Increasing evidence that bats actively forage at wind turbines
Source: PeerJ. 2017 Nov 3;5:e3985. doi: 10.7717/peerj.3985 (PMC5672837; doi:10.7717/peerj.3985)
Supplement: Figure S2 — Sampling curve used to determine how many clones were needed to detect the insects in (A) bat stomach samples (n = 68 stomachs) and (B) bat fecal pellets (n = 23 fecal pellets) collected at wind turbines at the Wolf Ridge wind farm. The number above each data point indicates the number of samples (stomachs or fecal pellets, respectively) contributing to each. [file peerj-05-3985-s002.docx]

| A | **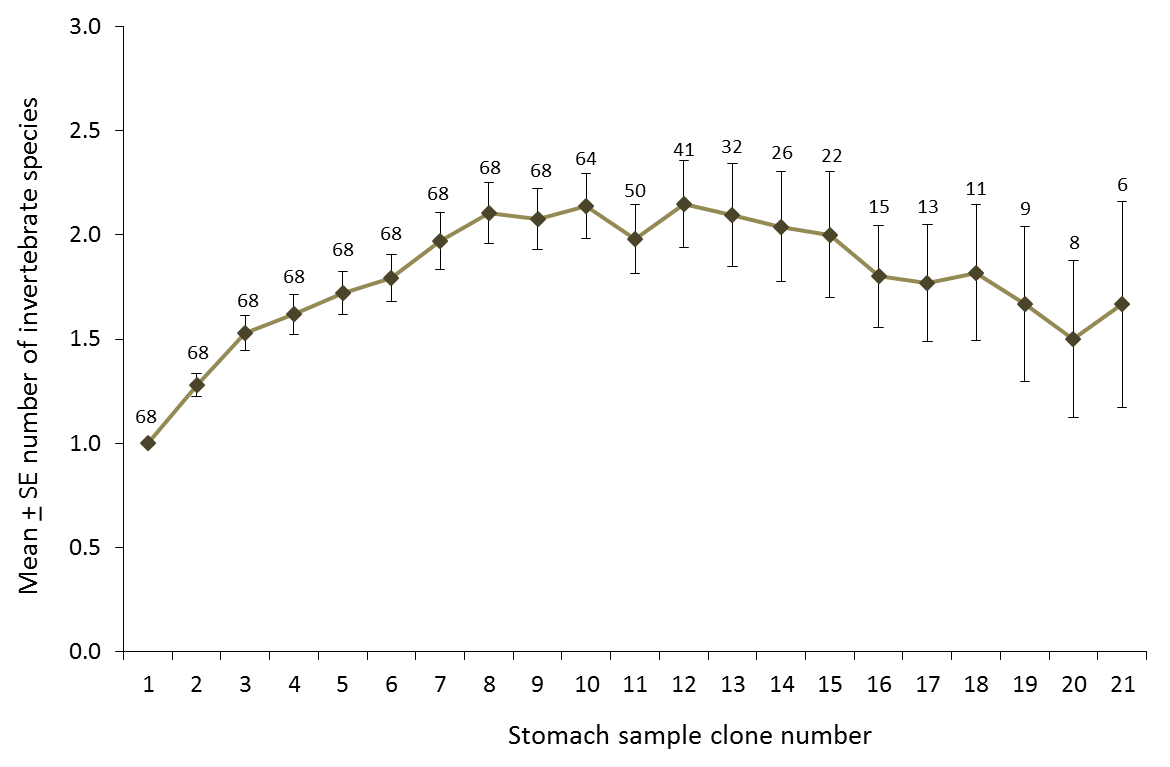** |
| --- | --- |
| B | **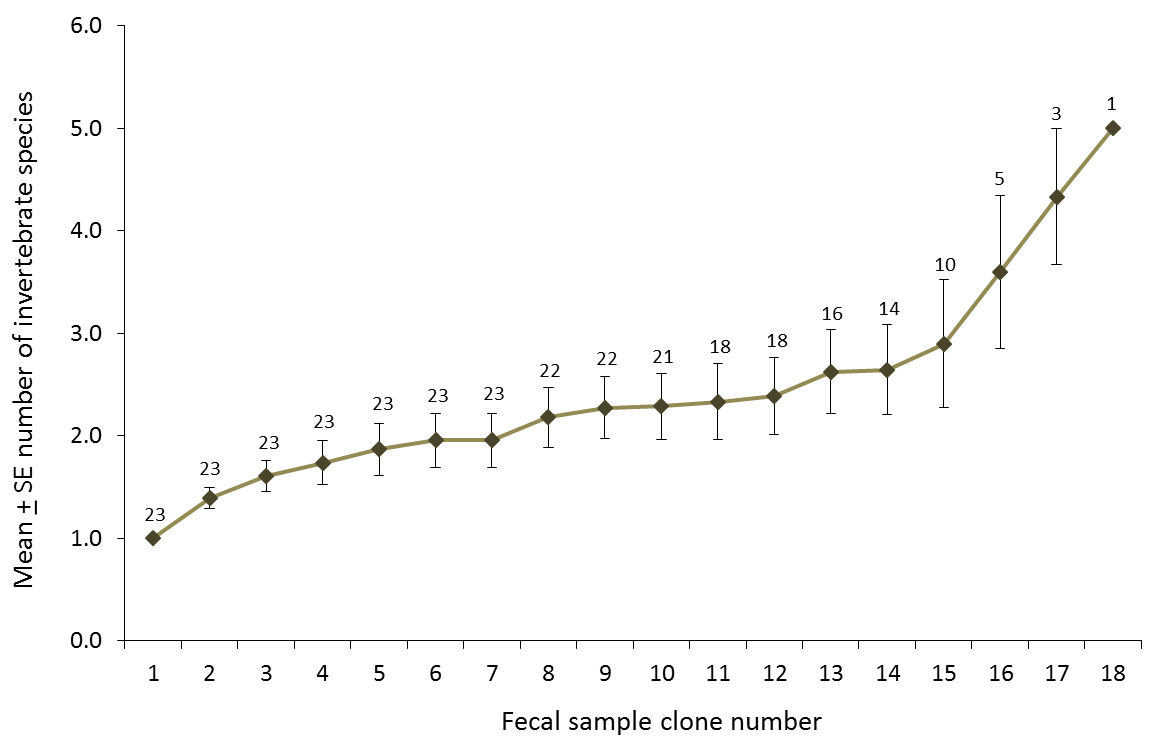** |

**Figure S2. Sampling curves for insect species detected in bat stomachs and fecal pellets.**

Sampling curve used to determine how many clones were needed to detect the insects in (A) bat stomach samples (n = 68 stomachs) and (B) bat fecal pellets (n = 23 fecal pellets) collected at wind turbines at the Wolf Ridge wind farm. The number above each data point indicates the number of samples (stomachs or fecal pellets, respectively) contributing to each.
