# Supplementary material for: Increasing evidence that bats actively forage at wind turbines
Source: PeerJ. 2017 Nov 3;5:e3985. doi: 10.7717/peerj.3985 (PMC5672837; doi:10.7717/peerj.3985)
Supplement: Figure S3 — Detection frequency of each insect species in eastern red bat stomachs (n = 45), eastern red bat fecal pellets (n = 23), and hoary bat stomachs (n = 23) collected from the Wolf Ridge wind farm. [file peerj-05-3985-s003.docx]

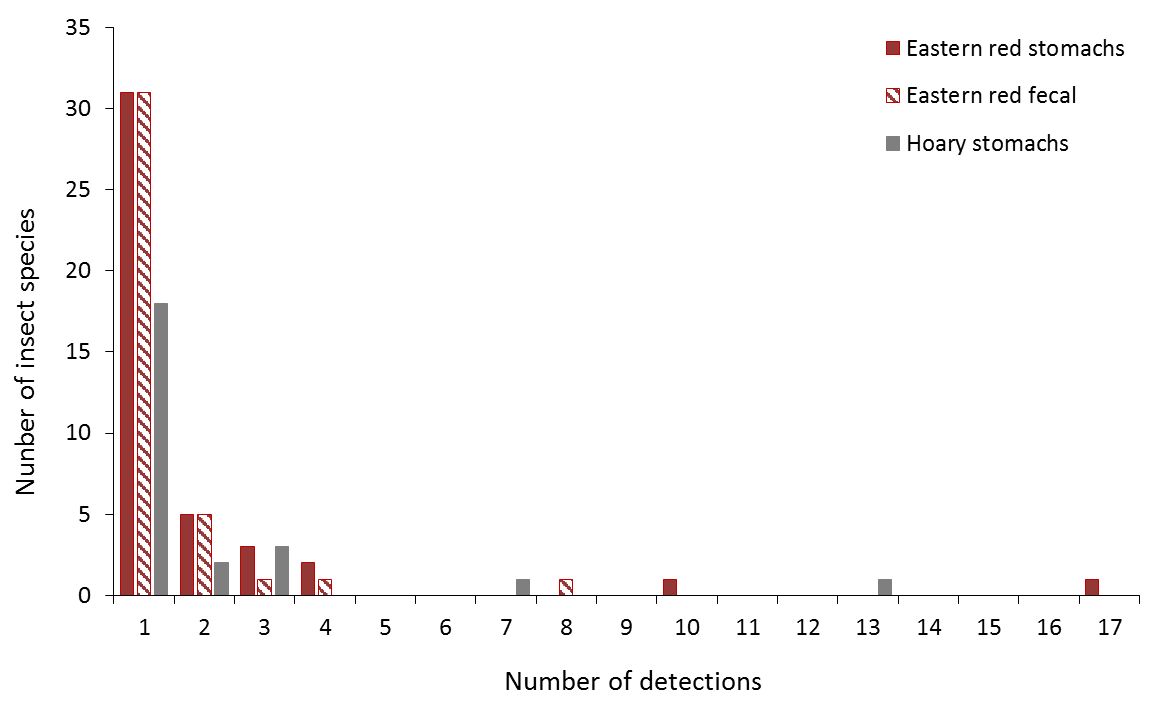


**Figure S3. Detection frequency of insect species in bat stomachs and fecal pellets.**

Detection frequency of each insect species in eastern red bat stomachs (n = 45), eastern red bat fecal pellets (n = 23), and hoary bat stomachs (n = 23) collected from the Wolf Ridge wind farm.
