# Supplementary material for: Increasing evidence that bats actively forage at wind turbines
Source: PeerJ. 2017 Nov 3;5:e3985. doi: 10.7717/peerj.3985 (PMC5672837; doi:10.7717/peerj.3985)
Supplement: Table S1 — Insects identified in eastern red bat stomachs collected from the Wolf Ridge wind farm in 2013 and 2014. Species identification is based on the percentage match in BOLD. Insects not identified to species in BOLD are differentiated by letters. Insects identified in eastern red bats collected in both years are indicated by (∗). [file peerj-05-3985-s004.docx]

**Table S1.** Insects identified in eastern red bat stomachs collected from the Wolf Ridge wind farm in 2013 and 2014. Species identification is based on the percentage match in BOLD. Insects not identified to species in BOLD are differentiated by letters. Insects identified in eastern red bats collected in both years are indicated by (*).

| **Order** | **Species** | **Number of stomachs** |
| --- | --- | --- |
| Blattodea | *Parcoblatta A** | 4 |
| Coleoptera | *Typhaea stercorea* | 1 |
|  | *Nitidulidae A* | 1 |
| Diptera | *Drosophila suzukii* | 1 |
|  | *Mycetophilidae A* | 1 |
| Hemiptera | *Hemiptera A* | 1 |
|  | *Perigenes A* | 1 |
| Lepidoptera | *Achyra rantalis** | 3 |
|  | *Acrolophus texanella* | 1 |
|  | *Argyrostrotis anilis* | 1 |
|  | *Bleptina caradrinalis** | 3 |
|  | *Bleptina n. sp. 4* | 1 |
|  | *Bulia deducta* | 1 |
|  | *Caenurgia chloropha* | 1 |
|  | *Clostera inclusa* | 1 |
|  | *Cydia latiferreana* | 1 |
|  | *Digrammia continuata* | 1 |
|  | *Elaphria A* | 2 |
|  | *Elaphria grata* | 1 |
|  | *Euchromius ocelleus* | 4 |
|  | *Fascista A* | 1 |
|  | *Gretchena bolliana* | 1 |
|  | *Helicoverpa zea* | 3 |
|  | *Homoeosoma electella* | 1 |
|  | *Idia concisa* | 2 |
|  | *Lepidoptera C* | 1 |
|  | *Lepidoptera D* | 1 |
|  | *Lepidoptera E* | 1 |
|  | *Lepidoptera G* | 1 |
|  | *Lepidoptera J* | 2 |
|  | *Melipotis jucunda* | 2 |
|  | *Ostrinia penitalis* | 1 |
|  | *Pelochrista A* | 1 |
|  | *Peoria tetradella* | 2 |
|  | *Peridroma saucia* | 1 |
|  | *Spodoptera frugiperda* | 11 |
|  | *Tripudia quadrifera* | 1 |
| Neuroptera | *Chrysoperla rufilabris* | 1 |
|  | *Myrmeleontidae A* | 1 |
| Orthoptera | *Allonemobius A* | 1 |
|  | *Allonemobius fasciatus* | 1 |
|  | *Gryllus spp.** | 29 |
|  | *Syrbula admirabilis* | 1 |
