# Supplementary material for: Increasing evidence that bats actively forage at wind turbines
Source: PeerJ. 2017 Nov 3;5:e3985. doi: 10.7717/peerj.3985 (PMC5672837; doi:10.7717/peerj.3985)
Supplement: Table S2 — Insects identified in hoary bat stomachs collected from the Wolf Ridge wind farm in 2013 and 2014. Species identification is based on the percentage match in BOLD. Insects not identified to species are differentiated by letters. Insects identified in hoary bats collected in both years are indicated by (∗). [file peerj-05-3985-s005.docx]

**Table S2.** Insects identified in hoary bat stomachs collected from the Wolf Ridge wind farm in 2013 and 2014. Species identification is based on the percentage match in BOLD. Insects not identified to species are differentiated by letters. Insects identified in hoary bats collected in both years are indicated by (*).

| **Order** | **Species** | **Number of stomachs** |
| --- | --- | --- |
| Coleoptera | *Digitonthophagus gazella* | 1 |
|  | *Hydrophilus triangularis* | 1 |
|  | *Lobopoda A* | 1 |
|  | *Thermonectus nigrofasciatus* | 1 |
| Lepidoptera | *Bleptina caradrinalis* | 3 |
|  | *Caenurgia erechtea* | 1 |
|  | *Condica sutor* | 1 |
|  | *Copitarsia n. sp. 1 RBS-2008* | 1 |
|  | *Cucullia laetifica* | 1 |
|  | *Depressaria alienella* | 1 |
|  | *Elaphria A* | 2 |
|  | *Euchromius ocelleus** | 3 |
|  | *Eudonia A* | 1 |
|  | *Galgula partita* | 1 |
|  | *Grammia arge* | 1 |
|  | *Helicoverpa zea** | 2 |
|  | *Hypena scabra* | 1 |
|  | *Lepidoptera A* | 1 |
|  | *Lepidoptera B* | 1 |
|  | *Lepidoptera I* | 1 |
|  | *Melipotis jucunda* | 1 |
|  | *Peridroma saucia* | 3 |
|  | *Spodoptera frugiperda** | 7 |
|  | *Zale A* | 1 |
| Orthoptera | *Gryllus rubens** | 18 |
