# Supplementary material for: Increasing evidence that bats actively forage at wind turbines
Source: PeerJ. 2017 Nov 3;5:e3985. doi: 10.7717/peerj.3985 (PMC5672837; doi:10.7717/peerj.3985)
Supplement: Table S3 — Insects identified in eastern red bat fecal pellets collected from the Wolf Ridge wind farm in 2011–2012. Species identification is based on the percentage match in BOLD. Insects not identified to species in BOLD are differentiated by letters. Insects identified in fecal pellets in >1 year are indicated by (∗). [file peerj-05-3985-s006.docx]

**Table S3.** Insects identified in eastern red bat fecal pellets collected from the Wolf Ridge wind farm in 2011-2012. Species identification is based on the percentage match in BOLD. Insects not identified to species in BOLD are differentiated by letters. Insects identified in fecal pellets in >1 year are indicated by (*).

| **Order** | **Species** | **Number of fecal pellets** |
| --- | --- | --- |
| Blattodea | *Parcoblatta A* | 1 |
| Coleoptera | *Anisodactylus merula* | 1 |
|  | *Hypera postica* | 1 |
|  | *Stegobium paniceum* | 4 |
|  | *Tenebrio molitor* | 3 |
|  | *Zygogramma suturalis* | 1 |
| Diptera | *Agromyzidae A* | 1 |
|  | *Cecidomyiidae A* | 1 |
|  | *Diptera A* | 1 |
|  | *Diptera B* | 1 |
|  | *Diptera C* | 1 |
|  | *Ephydridae A* | 2 |
|  | *Eurosta A* | 1 |
|  | *Eurosta B* | 1 |
|  | *Eurosta solidaginis* | 2 |
|  | *Stratiomyidae A* | 1 |
| Ephemeroptera | *Caenis A* | 1 |
| Hemiptera | *Hemiptera C* | 1 |
| Isopoda | *Armadillium A* | 1 |
| Lepidoptera | *Agrotis malefida* | 1 |
|  | *Elaphria A* | 1 |
|  | *Euptoieta A* | 1 |
|  | *Geometridae A* | 1 |
|  | *Homoeosoma electella* | 1 |
|  | *Lepidoptera F** | 2 |
|  | *Lepidoptera H* | 1 |
|  | *Nathalis iole* | 1 |
|  | *Orthosia alurina* | 1 |
|  | *Peridroma saucia** | 2 |
|  | *Phoberia A* | 1 |
|  | *Pontia protodice* | 1 |
|  | *Rachiplusia ou* | 1 |
|  | *Spodoptera frugiperda* | 2 |
|  | *Strymon melinus* | 1 |
| Neuroptera | *Chrysoperla rufilabris* | 1 |
|  | *Sympherobius A* | 1 |
| Orthoptera | *Gryllus A** | 8 |
|  | *Melanopus bivittatus* | 1 |
|  | *Spharagemon collare* | 1 |
